# Age Differences in Social Discounting and Charitable Giving in a U.S. Sample

**DOI:** 10.1093/geroni/igaf122.1876

**Published:** 2025-12-31

**Authors:** Yi Lu, Corinna Löckenhoff

**Affiliations:** Cornell University, Ithaca, New York, United States; Cornell University, Ithaca, New York, United States

## Abstract

Social discounting describes the tendency to show less generosity as social distance to the recipient increases. Prior studies examining age differences in social discounting suggest that older age is associated with greater generosity toward distant others (Pornpattananangkul et al., 2019; Gong et al., 2019). However, the generalizability of these findings is limited due to small sample sizes, comparisons of extreme age groups, and an exclusive focus on Asian samples. To address these gaps, a pre-registered study examined age differences in social discounting and charitable giving in a U.S. adult life-span sample (N = 432, aged 18-93, Mage = 51.23, SDage = 18.92, 50% female, 55% non-Hispanic White). We predicted that older age would be associated with lower social discounting and increased generosity across social distances and towards charities. The social discounting measure was adapted from Jones and Rachlin (2006) and involved five social distances (1/5/10/50/100, ranging from 0 = closest, 100 = most distant). For each social distance, participants chose between giving $100 to the other person and receiving $0/$20/$40/$60/$80/$100/$120 for themselves alone. They made the same decision for their favorite charity. Contrary to predictions, there were no significant age differences in social discounting rates, generosity across social distances, and charitable giving (ps >.05). Regardless of age, charitable giving was positively associated with generosity across social distances (ρ = .39, p < .001). This pattern of effects remained robust after controlling for demographics. Findings suggest that age differences in social discounting may be sensitive to cultural setting and methodological factors.

